# Carotid Stump Syndrome With Stent-Assisted Coil Embolization

**DOI:** 10.7759/cureus.22746

**Published:** 2022-03-01

**Authors:** Cordell Baker, Brandon Sherrod, Nicholas T Gamboa, Philipp Taussky, Ramesh Grandhi

**Affiliations:** 1 Neurosurgery, University of Utah, Salt Lake City, USA

**Keywords:** carotid stump, stroke, carotid sacrifice, stent-assisted coil embolization, endovascular procedures, carotid stump syndrome

## Abstract

Carotid stump syndrome (CSS) is a rare cause of recurrent ipsilateral cerebrovascular events that typically manifests as transient ischemic attacks or amaurosis fugax. The cause of these recurrent symptoms is thought to be microembolization from an occluded internal carotid artery that reaches intracranial circulation through anastomoses. We undertook a systematic literature review according to Preferred Reporting Items for Systematic Reviews and Meta-Analyses (PRISMA) guidelines using the PubMed, Web of Science, and Embase databases of the endovascular treatment options for CSS. Nine papers met the inclusion criteria and provided patient data on 12 patients, and one case illustration is presented. Treatment was with common carotid artery-external carotid artery stent graft without concomitant coil embolization in nine patients and with coil embolization without stenting, the breakthrough of the stump with a wire and subsequent internal carotid artery stent placement, and stent-assisted coil embolization in one patient each. During a median follow-up of six months, all patients were on dual antiplatelet therapy except one on undefined “systemic anticoagulation.” Twelve patients had no symptoms after treatment, one had transient expressive aphasia but no further symptoms after being placed on anticoagulation, and none had intraprocedural complications or had to undergo retreatment. Our review indicates that endovascular treatment of CSS is associated with low intraprocedural risk and is effective at treating recurrent symptoms.

## Introduction

Carotid stump syndrome (CSS) is defined as persistent cerebrovascular events due to complete occlusion of an ipsilateral internal carotid artery (ICA) [1-4]. There are multiple theories regarding the etiology of these events, but many believe the syndrome is a result of microembolizations from the internal carotid stump into the external carotid artery (ECA) that enters intracranial circulation via ECA-to-ICA anastomoses [3, 5, 6]. CSS is a relatively rare entity, but if not diagnosed and treated properly, it can lead to recurrent symptoms and neurologic impairment [1, 3].

CSS was traditionally treated with the open surgical exclusion of the stump and endarterectomy of the ECA [7]. More recently, endovascular treatments involving common carotid artery (CCA)-to-ECA stenting have gained popularity [3, 8, 9]. Despite multiple case reports and small series of endovascular treatments for CSS, there has been no review of the existing literature to establish the efficacy and safety of endovascular intervention for the treatment of CSS.

In this study, we present a systematic review of endovascular treatments for CSS and provide a case illustration of carotid stump exclusion with stent-assisted coil embolization. To our knowledge, this is the second study to describe stent-assisted coil embolization for CSS [8] and the only study to review the literature in its entirety and compile the endovascular data among these patients.

## Case presentation

**Figure 1:**
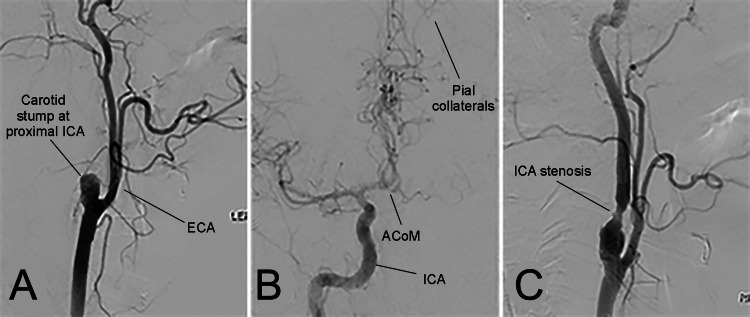
Digital subtraction angiography confirming left internal carotid artery (ICA) occlusion with a stump. Images showing left ICA stump (A), good pial collaterals with filling contralateral anterior circulation filling through the anterior communicating artery (ACoM), absence of filling of the proximal M1 segment (B), and right ICA stenosis of 70% (C). ECA, external carotid artery.

A 58-year-old man with nasopharyngeal carcinoma who had a resection and postoperative radiation and cochlear implant with residual speech difficulty was transferred to our hospital with acute-onset, right-side weakness, and worsening speech from baseline. His National Institutes of Health Stroke Scale score was seven. Computed tomography angiography revealed an extracranial left ICA occlusion and an intracranial left middle cerebral artery (MCA) M1 segment occlusion. He was taken emergently for a digital subtraction angiogram, which confirmed the left ICA occlusion with a stump (Figure 1) as well as a left M1 occlusion. However, he appeared to have excellent collateral circulation with a filling of the left anterior circulation via cross filling across the anterior communicating artery and pial-pial collaterals from the left anterior and posterior cerebral artery territories to the left MCA territory. In addition, he was found to have 70% stenosis of the extracranial right ICA. He was started on dual antiplatelet therapy with aspirin and clopidogrel. After transcranial Doppler ultrasound (TCD) revealed evidence of emboli in the left MCA, the patient was discharged on aspirin and apixaban. The most likely explanation for positive TCD emboli in the left MCA distribution despite initial M1 segment occlusion is that the M1 segment was reconstituted after the initial digital subtraction angiography (DSA) and symptom onset. He returned to our hospital two weeks later with clinical evidence of a right-sided anterior circulation stroke. Prior to stenting the right ICA, we discontinued the apixaban and started the patient on prasugrel. Following the stenting procedure, TCDs once again revealed emboli in the left MCA. We discussed medical management consisting of antithrombotic therapy with aspirin, prasugrel, and apixaban versus performing stent-assisted coiling of the left carotid stump with the patient and his family. They wished to obviate the need for triple antithrombotic therapy and to proceed with left carotid stump embolization.

**Figure 2:**
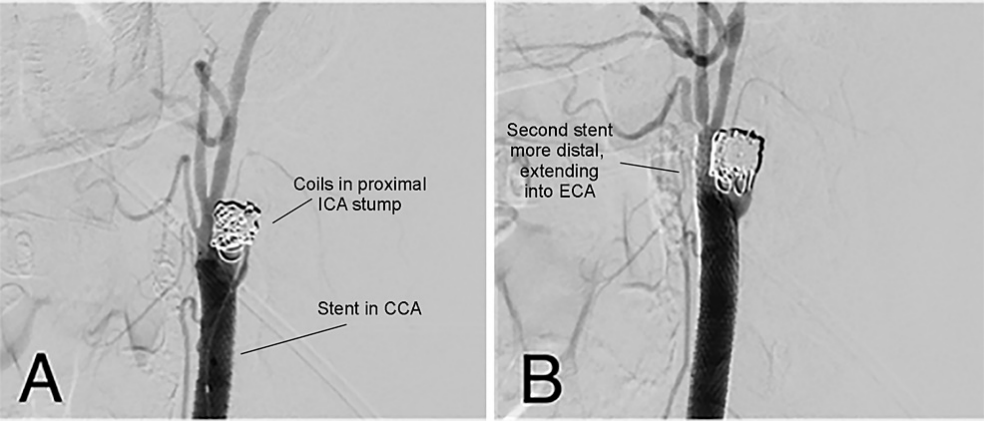
Digital subtraction angiography after placement of coils. (A) Image showing stent-assisted coil embolization of the carotid stump. (B) A second stent was deployed to ensure that the stent covered the entirety of the bifurcation. The stump shows reduced filling when compared with the original angiogram. CCA, common carotid artery; ECA, external carotid artery; ICA, internal carotid artery.

A 9F Cello balloon guide catheter (Medtronic, Irvine, CA, USA) was placed in the left CCA. Intravenous heparin was administered to target an activated clotting time of 225 to 250. An Aristotle-14 microwire (Scientia Vascular, Salt Lake City, UT, USA) was advanced into the left ECA under roadmap guidance. Next, under circulatory flow arrest, two telescoping 6×40 mm Precise stents (Cordis, Santa Clara, CA, USA) were placed from the left ECA to the proximal left CCA. After placement of the stents, a Prowler Select LP Microcatheter (Cerenovus, Irvine, CA USA) was advanced over an Aristotle-14 microwire into the left ICA stump. Coiling of the left ICA stump was performed through the Prowler Select LP Microcatheter using Optima (BALT, Irvine, CA, USA). Following the placement of the coils, the microcatheter was removed. DSA showed decreased flow within the carotid stump, with patency of the left ECA-to-CCA stent (Figure 2).

There were no complications following the procedure. Subsequent TCD showed no evidence of thromboemboli in the left anterior circulation. The patient was discharged on dual antiplatelet therapy. At the one-year follow-up, the patient remains at his neurologic baseline with no new stroke symptoms.

## Discussion

Systematic literature review of carotid stump syndrome and endovascular treatment

We performed a systematic review of data with the outcome of interest being carotid stump syndrome with endovascular treatment. The results are presented individually as cases as this is a relatively rare entity treated with heterogeneous modalities. Variables of interest included intraoperative complications, need for additional treatment, and recurrence of stroke-like symptoms at longest follow-up interval.

**Figure 3:**
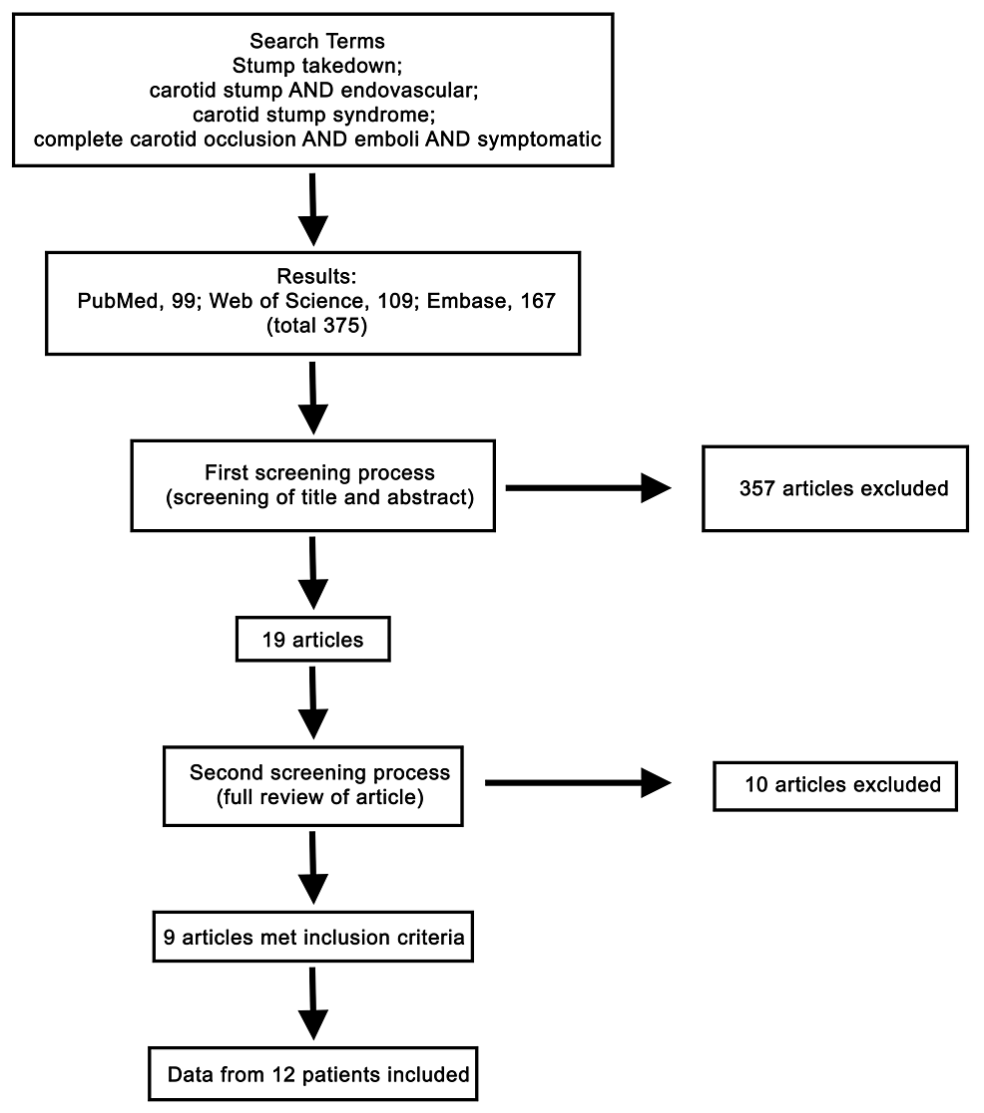
Flow diagram showing the combination of search terms used to complete the search and the results from each database and screening.

The study was designed according to the preferred reporting items for systematic reviews and meta-analyses guidelines for systematic reviews. The review consisted of a literature search using the PubMed, Web of Science, and Embase databases. Each database was searched using the combinations of terms found in Figure 3. Every article was screened for inclusion by reading the title and abstract. If the article did not meet the inclusion criteria based on initial review, the article was excluded; articles that were not excluded were included in a list of articles to be read in their entirety during a second screening process. The inclusion and exclusion criteria for the screening processes are found in Table 1.

**Table 1:** Inclusion and exclusion criteria for screening process ICA: internal carotid artery

Inclusion Criteria	Exclusion Criteria
Age > 17 years	Age < 17 years
Diagnosis of carotid stump syndrome	Complete ICA occlusion but asymptomatic
Neurologic symptoms from ipsilateral ICA that is occluded	Symptoms not consisted with stump emboli
All endovascular treatments	Treatment with surgery
	Treatment with only medical management

The first literature review resulted in 375 articles. These were found by using all combinations of search terms in the databases (Pubmed, 99; Web of Science, 109; Embase, 167). After the abstracts and titles from the 375 articles were reviewed, 19 articles passed the initial screening process. These 19 articles were read in their entirety, and 10 more articles were excluded. Article exclusion was due to patients who underwent nonendovascular (either surgical or medical) treatments, patients reported as having CSS but who were asymptomatic or whose deficits were not consistent with emboli from the ipsilateral side of occlusion, and patients <18 years old. In total, nine papers met the inclusion criteria and provided patient data on 12 patients (Figure 3) [3, 8-15].

Data summary

Including our case, 13 patients were included in the report (Table 2). The median age was 68. Two patients were < 40-years-old; one was diagnosed with CSS due to an arterial dissection and the other had congenital agenesis of the ICA. Ten patients were male. Six patients had left-sided pathology and one patient had bilateral CSS. Nine patients (69%) underwent treatment with CCA-ECA stent-graft without concomitant coil embolization. One patient was treated with coil embolization without stenting, one patient had a breakthrough of the stump with a wire and subsequent ICA stent placement, and two patients were treated with stent-assisted coil embolization.

**Table 2:** Patient data from publications that met the inclusion criteria –, not available; CSS: carotid stump syndrome; DAPT: dual antiplatelet therapy; mo: months; wks: weeks; M: male; F: female; R: right; L: left; LUE: left upper extremity; LLE: left lower extremity; BL: bilateral; Y: yes; N: no; CCA: common carotid artery; ECA: external carotid artery; ICA: internal carotid artery; MI: myocardial infarction; post-op: postoperatively

Patient no.	Reference	Age/ sex	CSS side	Symptoms	Time from symptom onset to treatment	Endovascular treatment	Type of DAPT, if known	Last known follow-up (mo)	Symptom recurrence & complications	Follow-up imaging
1	Naylor et al. 2003 [14]	61M	L	R side numbness; speech difficulty	2 mo	CCA–ECA stent graft	“Systemic anticoagulation”, not specified	3	Transient expressive aphasia, none after anticoagulation use	Patent stent
2	Nano et al. 2006 [13]	71F	R	L side weakness, numbness	6 mo	CCA–ECA stent graft	aspirin, ticlopidine	6	N	Patent stent
3	Carrafiello et al. 2009 [9]	72F	R	L side weakness	-	CCA–ECA stent	aspirin, clopidogrel	12	N	–
4	Lakshminarayan et al. 2010 [3]	52M	R	amaurosis fugax, LUE numbness	-	CCA–ECA stent graft	aspirin, clopidogrel	60	N	Patent stent
5	Lakshminarayan et al. 2010 [3]	67F	L	R side weakness	24 mo	CCA–ECA stent graft	-	36	N	Stent occlusion
6	Lakshminarayan et al. 2010 [3]	70M	R	amaurosis fugax	-	CCA–ECA stent graft	-	12	N	Patent stent
7	Dakhoul and Tawk 2014 [10]	–M	L	amaurosis fugax	1 mo	CCA–ECA stent	-	6	N	-
8	Shin et al. 2015 [8]	39M	L	R side weakness	-	CCA–ECA stent, coil embolization	triflusal, clopidogrel	5	N	-
9	Mahajan et al. 2018 [12]	36M	L	R side numbness, speech difficulty	-	Coil embolization of stump w/out stent	aspirin, clopidogrel	6	N	-
10	Dulai et al. 2018 [11]	68M	R	amaurosis fugax	9 mo	CCA–ECA stent	“placed on best medical management”	36	N	Stent occlusion
11	Dualai et al. 2018 [11]	72M	BL	BL amaurosis fugax	2 mo	BL CCA–ECA stents	-	1	No symptom recurrence, MI 4-week post-op with complete recovery	-
12	Xu et al. 2019 [15]	71M	R	LLE weakness	3 mo	ICA stent	aspirin, clopidogrel	9	N	Patent stent
13	This paper	58M	L	R side weakness, speech difficulty	2 wks	CCA–ECA stent, coil embolization	aspirin, prasugrel	1	N	-

The median follow-up time was six months (range one to 60 months). All but one patient was placed on dual antiplatelet therapy, one report stated that the patient was placed on “systemic anticoagulation” but did not specify the medication(s) or if the patient was on antiplatelet therapy. Twelve of 13 patients had no symptoms after treatment. One patient had transient expressive aphasia but no further symptoms after being placed on anticoagulation. None of the patients had intraprocedural complications or had to undergo retreatment.

Endovascular treatment of carotid stump syndrome

**Figure 4:**
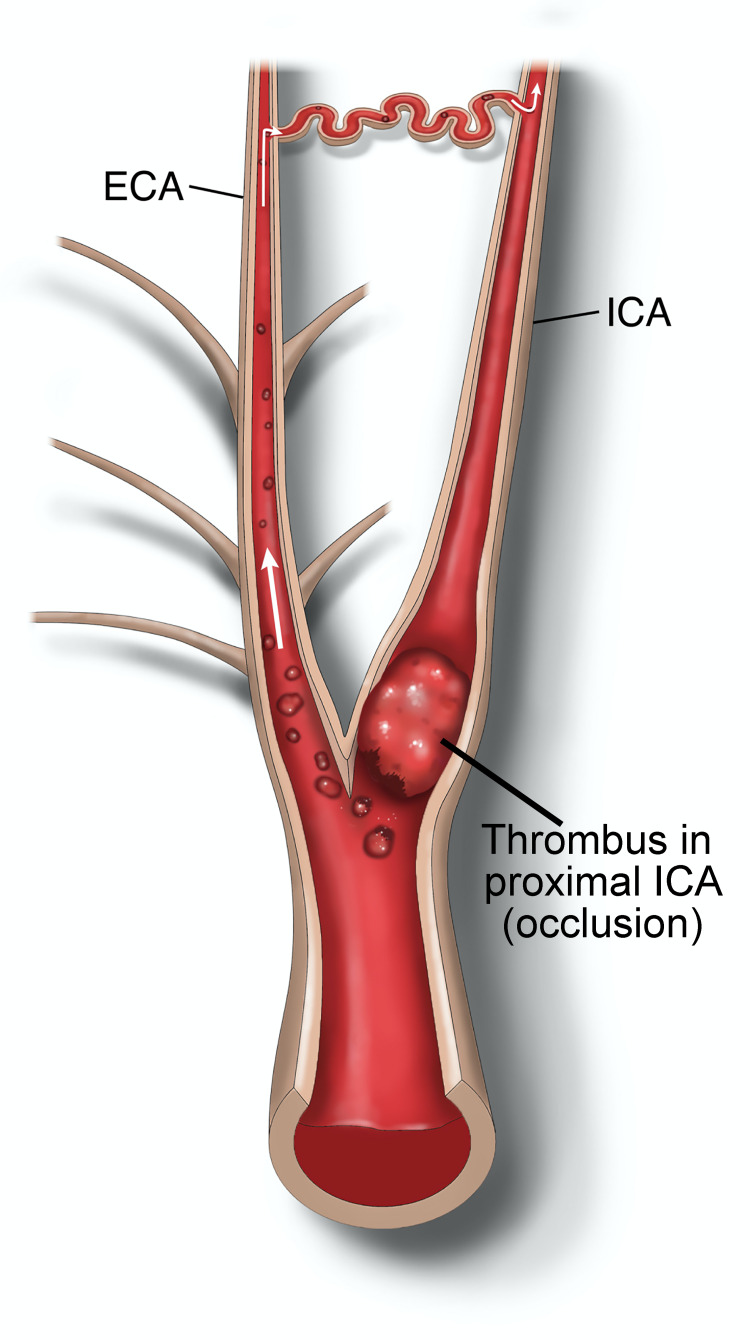
Illustration of the etiology of recurrent ischemic attacks in carotid stump syndrome. Emboli from the internal carotid artery stump travel through the external carotid artery and return to the intracranial circulation through the distal external carotid artery–to–internal carotid artery anastomoses.

Recurrent transient ischemic attacks in patients with ipsilateral complete ICA occlusion were first described in 1976 [14]. In patients with complete ICA occlusion, a proportion has a small proximal ICA segment before the occlusion that continues to fill, commonly referred to as the “stump.” The recurrent ischemic events associated with the stump are known as “stump syndrome.” The etiology of these symptoms was thought to be due to either microembolization from the carotid stump versus hypoperfusion of the occluded side. However, efforts to treat hypoperfusion of the occluded side with extracranial-intracranial bypass did not result in symptom improvement [16]. Thus, microembolization from the stump became the favored hypothesis. There are multiple theories on the source of emboli in these patients, including microembolization propagating intracranially from the distal portion of the ICA stump and intracranial microembolization through distal ICA-ECA anastomotic channels (Figure 4) [5, 6]. The latter is thought to be the most probable cause of symptoms and is the most heavily cited explanation. Further evidence of microembolization and not hypoperfusion as the source of symptoms is based on TCD emboli detection, which has confirmed that emboli from the stump pass into intracranial circulation [17].

Before the advent of endovascular treatments, many patients with CSS were treated with anticoagulation therapy or open surgery [7, 11, 18]. The latter involves performing an ECA endarterectomy with obliteration of the ICA stump with oversewing or with the placement of a large metallic clip. The largest study to date [7] of patients who underwent open treatment for CSS includes 25 patients; all but one of whom remained asymptomatic at follow-up. One patient experienced an internal capsule infarct and two patients developed postoperative cranial nerve deficits. In a pilot study comparing best medical management and surgical treatment in patients with CSS and one symptomatic event, 10 patients were randomized to medical therapy and 15 patients to surgery [19]. One vascular event recurred in a patient who was treated by medical means. There was no mention in this study of intraoperative or postoperative complications unrelated to stroke.

Another study compared the outcomes of open versus endovascular treatment for CSS [11]. Five patients were treated with open surgery and four patients with a CCA-ECA covered stent. All patients had resolution of symptoms at follow-up. One patient in the stent cohort sustained a myocardial infarction 4 weeks after intervention [11]. The authors concluded that both open and endovascular treatments were safe and effective. Of note, only two of the four patients in the stent cohort were included in our study, as the presenting symptoms of the other patients were not consistent with ipsilateral neurologic events.

In our systematic review, a total of 13 patients underwent endovascular treatment for CSS. None of the patients experienced an intraoperative complication. One patient had transient expressive aphasia seven days after stent placement but returned to his neurologic baseline after being placed on anticoagulation therapy [14]. The majority of patients had endovascular treatment with a covered stent. Besides our case, there was only one other reported case of stent-assisted coil embolization of a carotid stump [8]. Similar to the studies on open surgery, the majority of patients who underwent endovascular treatment did not have symptom recurrence. Two of the 13 patients developed complete occlusion of the stent on follow-up imaging but remained asymptomatic [3, 11]. These patients may have developed excellent collaterals from the contralateral circulation and thus were not reliant upon perfusion from the ipsilateral ECA.

A direct comparison of endovascular versus open surgical treatment of CSS is difficult to make because of the limited number of cases and heterogeneity of the patient population. An additional confounder is that endovascular treatment is continuously evolving, and outcomes from past treatments may not be the same as current outcomes. Despite the difficulties in comparing these groups, the reported cases from both treatment modalities had relatively favorable outcomes. In our endovascular review, no patients experienced major intraoperative complications, and only one patient had a major postoperative complication with a myocardial infarction four weeks after treatment (from which he fully recovered). Most patients did not experience a complication in the open surgical series, although one of 25 patients had a major stroke and two patients developed cranial nerve palsies [7]. There was no reported recurrence of vascular symptoms in the surgical groups reviewed [7, 11, 19]. There were no recurrences of symptoms in these data from the endovascularly treated patients who were discharged with medical management, one patient had a recurrence of symptoms but none after being placed on medical therapy. 

CSS is a rare entity and thus there is a paucity of data on outcomes of endovascular treatment. We focused the search terms to focus the identified papers on carotid stump syndrome with endovascular treatment. We acknowledge that we might have missed identifying pertinent papers that could have been revealed with broader searches. It should also be noted that case reports and small case series are typically composed of favorable outcomes and may not include single cases with complications. It is also difficult to analyze these data as one cohort because the modality of endovascular treatment was heterogeneous across studies. The distinction should also be made that not all patients likely require CCA-ECA stent with coil embolization of the ICA and that treatment with coil embolization alone may suffice. In this patient, we thought it warranted based on the anatomy and fear of coil extrusion with consequent potential for thromboembolic complication. We acknowledge these limitations but believe that reporting the outcomes associated with endovascular treatment of this rare condition is necessary to guide future management by providers.

## Conclusions

CSS is a rare cause of recurrent ipsilateral cerebrovascular events that are thought to be caused by microembolization from an occluded ICA that reaches intracranial circulation through anastomoses. The results of this systematic review indicate that endovascular treatment of carotid stump syndrome is effective at treating recurrent symptoms of a transient ischemic attack. During median six-month follow-up, all patients were on anticoagulation therapy, none had symptoms after treatment, and one had transient expressive aphasia, but no other complications were reported. ECA-to-ICA stenting with a covered stent or stent-coiling with a traditional carotid stent appears to be efficacious options that result in mitigation of dispersal of thromboembolic from the stump into the intracranial circulation. Although future studies with larger cohorts are needed to make a definitive conclusion on the effectiveness of endovascular treatment, the rarity of the syndrome makes this difficult.
